# Skin cooling and topical application of menthol can reduce propofol injection-induced pain, a randomized trial

**DOI:** 10.1097/MD.0000000000042809

**Published:** 2025-06-13

**Authors:** Takashi Ishida, Natsuko Watanabe, Satoshi Tanaka, Mikito Kawamata

**Affiliations:** a Department of Anesthesiology and Resuscitology, Shinshu University School of Medicine, Matsumoto City, Nagano, Japan.

**Keywords:** injection-induced pain, propofol, transient receptor potential melastatin 8

## Abstract

**Background:**

Skin cooling has an analgesic effect on propofol injection-induced pain. We evaluated the analgesic effectiveness of topical menthol application and skin cooling for reducing propofol injection-induced pain.

**Methods:**

Patients (n = 120) were randomized to a cooling group, a room temperature group (non-cooling group), a menthol group, and a solvent group. In the cooling group, a cooling gel pad (7°C) was applied to the forearm. In the non-cooling group, a gel pad (25°C) was used. In the menthol group, 30% menthol was applied to the forearm. In the solvent group, a solvent was applied. Fifteen minutes after these interventions, propofol was intravenously injected. We assessed the incidence and severity of propofol injection-induced pain.

**Results:**

The incidence of propofol injection-induced pain in the cooling group was significantly lower than that in the non-cooling group (27% vs 52%, *P* = .049). The pain intensity in the cooling group was lower than that in the non-cooling group (*P* = .027). The incidence of propofol injection-induced pain in the menthol group was significantly lower than that in the solvent group (13% vs 40%, *P* = .020). The pain intensity in the menthol group was lower than that in the solvent group (*P* = .021).

**Conclusion:**

Topical menthol application and skin cooling are effective for reducing propofol injection-induced pain.

## 1. Introduction

Peripheral veins are innervated with polymodal nociceptors^[1,2]^ and intravenous (IV) injection of certain anesthetic agents activates the polymodal nociceptors and causes pain.^[3]^ Propofol is one of the drugs that is known to cause intense burning pain during IV injection. Although many studies have suggested molecular and physiological mechanisms of venous pain,^[2–5]^ the mechanism of venous pain is not fully understood, and analgesic treatment based on the mechanism has not been established. A recent study has shown an analgesic effect of skin cooling on propofol injection-induced pain.^[6]^ Skin cooling is easy and safe and would be a preferable method for preventing propofol injection-induced pain. There are several possible analgesic mechanisms of skin cooling, including reduction of metabolic, electrogenic, and ionic activities in neural tissue and activation of inhibitory neurons,^[7,8]^ and activation of transient receptor potential melastatin 8 (TRPM8) is known to play an important role in the analgesic effect of skin cooling on neuropathic pain.^[9]^ TRPM8 is a receptor that is sensitive to a non-noxious mild cooling temperature (<28˚C).^[10,11]^ However, an analgesic effect of TRPM8 activation on propofol injection-induced pain has not been shown.

We examined first whether cooling of the skin to a temperature at which TRPM8 is activated can prevent propofol injection-induced pain and then evaluated whether topical application of a TRPM8 agonist (menthol) on the skin can prevent propofol injection-induced pain.

## 2. Methods

### 2.1. Subjects

**Figure 1.**
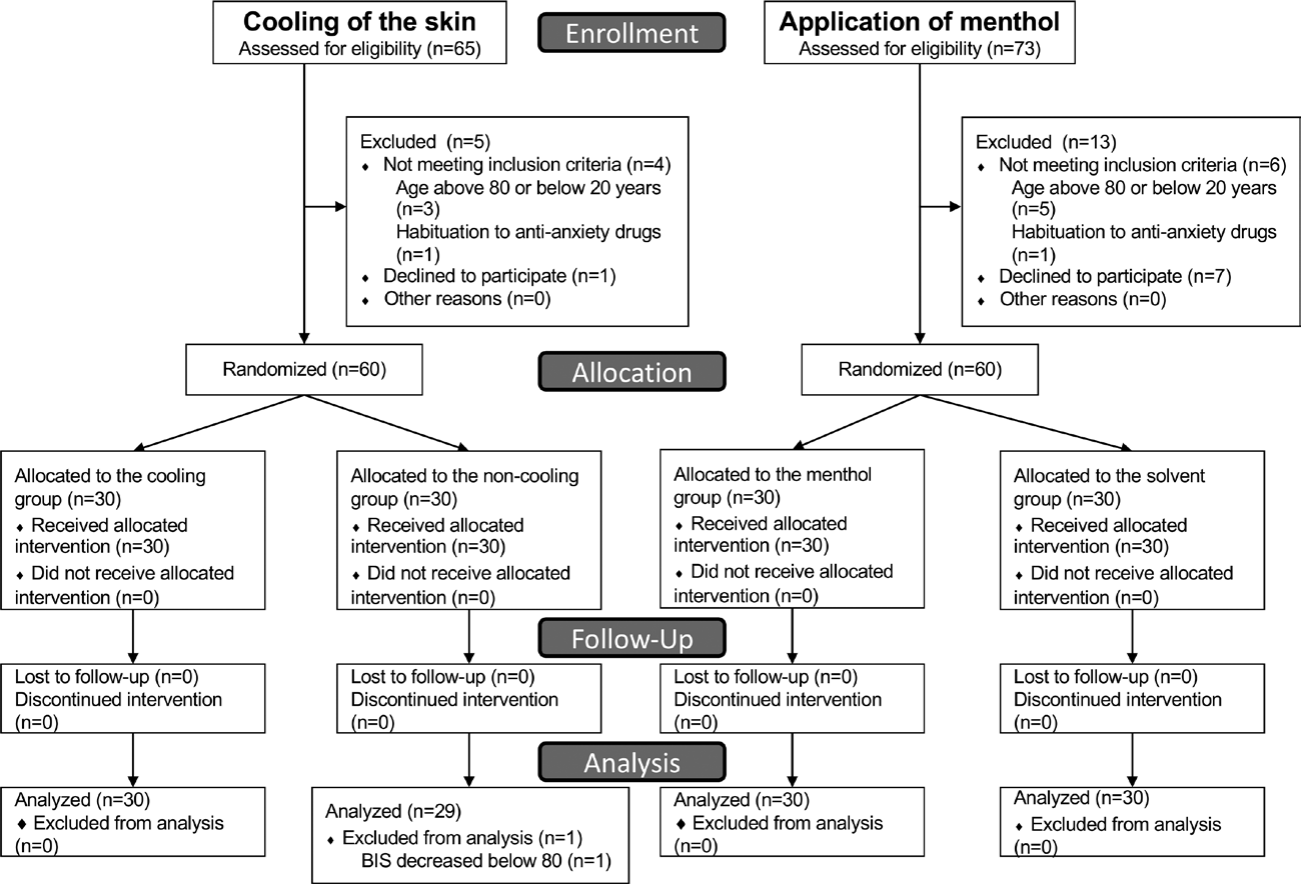
Consort flow diagram showing recruitment, allocation, follow-up, and analysis in the first part of the study (cooling of the skin) and the second part of the study (application of menthol).

This study consisted of 2 parts: analgesic effects of skin cooling and topical application of a TRPM8 agonist (menthol) on propofol injection-induced pain were evaluated in the first part and the second part, respectively. The study protocols were approved by the Institutional Ethics Committee of Shinshu University School of Medicine, Matsumoto, Japan (document number: 3340) on February 3, 2016. The study was then registered with the University Hospital Medical Information Network (UMIN) in Japan (ID: UMIN000021078) on February 17, 2016. This study was carried out in an operating theater of Shinshu University Hospital, Matsumoto, Japan. The first part was carried out from March to May in 2016 and the second part was carried out from June to September in 2016. Written informed consent was obtained from each patient enrolled in this study. One hundred twenty patients aged 20 to 80 years, American Society of Anesthesiologists physical status I or II, who were scheduled for elective surgery under general anesthesia were included in the 2 parts of the study (Fig. 1). Patients with vascular diseases, habituation to analgesics, sedatives or anti-anxiety drugs, allergic diseases or sensitivity to propofol, and infection on the dorsum of their left hands were excluded from the study.

### 2.2. Effect of cooling of the skin

Sixty patients were included in the first part of this study. In this part, the patients were randomly divided by a computer-generated random number list into a cooling group and a room temperature group (non-cooling group). We decided to use blocks of random sizes of 4. Neither the patient nor the anesthesiologist who performed interventions could be blinded. Only the assessor (NW) who assessed the pain was blinded to the group allocation.

**Figure 2.**
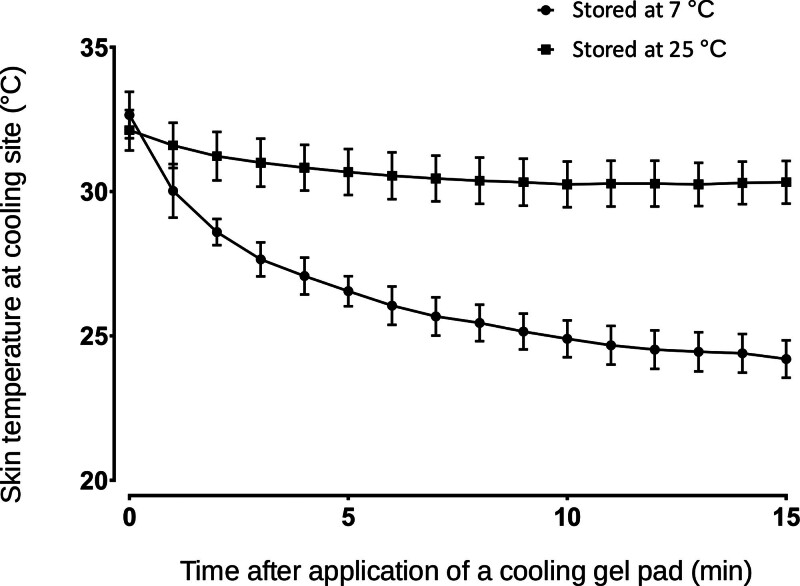
Skin temperature during interventions. Filled circles show skin temperature during application of a gel pad that had been stored at 7°C and filled squares show skin temperature during application of a gel pad that had been stored at 25°C. Data are shown as means and standard deviations.

No premedication was given. After standard monitoring (pulse oximetry, electrocardiogram, and noninvasive arterial pressure) and bispectral index (BIS) monitoring had been started, a 20-gauge IV catheter (SURFLO®, Terumo Co., Tokyo, Japan) was inserted into the dorsal vein of the left hand in all of the patients. Topical anesthesia was not used for insertion of the catheter. Acetated Ringer solution was infused at 200 mL/h. In the cooling group, a rectangular cooling gel pad (10 cm × 15 cm) that had been stored at 7°C was applied to the dorsum of the left forearm. The distal edge of the cooling gel pad was positioned at the wrist. In the non-cooling group, the cooling gel pad that had been stored at room temperature (25°C) was applied to the dorsum of the left forearm in the same way as was done in the cooling group. Prior to this study, we evaluated the effect of application of the cooling gel pad on skin temperature using 4 volunteers. A temperature probe with a diameter of 25 mm (STS-400, Smiths Medical Inc., Minneapolis, MN) was attached to the forearm of each volunteer and skin temperature was measured. Then the cooling gel pad that had been stored at 7°C or 25°C was placed on the temperature probe and the changes of skin temperature were recorded. Application of the cooling gel pad that had been stored at 7°C induced a cool sensation without eliciting cold pain and decreased the skin temperature of the forearm from 33 ± 1°C to 24 ± 0.6°C (Fig. 2). Application of the gel pad that had been stored at 25°C did not decrease skin temperature of the forearm below 30°C. We determined the optimal cooling temperature and cooling time to achieve the skin temperature that activates TRPM8 from the preliminary study. Fifteen minutes after these interventions, 0.5 mg/kg of propofol (Maruishi Co., Osaka, Japan) was injected into the vein through the IV catheter over a period of 5 seconds in both groups. No other analgesics or sedatives were administered before propofol injection.

One minute after the injection of propofol, the patients were asked standard questions regarding comfort of the injection. Outcomes evaluated were the incidence and intensity of propofol injection-induced pain according to a previously described method.^[12]^ The intensity of propofol injection-induced pain was assessed by using a 4-point numerical rating scale (NRS): none = 0 (negative response to questioning), mild pain = 1 (pain reported only in response to questioning with no behavioral signs), moderate pain = 2 (pain reported in response to questioning and accompanied by a behavioral sign or pain reported spontaneously without questioning), severe pain = 3 (strong vocal response or response accompanied by facial grimacing, arm withdrawal, or tears).^[12]^ The incidence of propofol injection-induced pain was defined as the incidence of the 4-point NRS of 1 or higher. Assessments were performed by 1 assessor (NW), who remained blinded to treatment allocation. Patients whose BIS decreased below 80 before questioning were excluded. After evaluation of the NRS scores, induction of anesthesia was continued with IV fentanyl at 2 to 3 μg/kg followed by the remainder of the calculated dose of propofol and administration of rocuronium at 0.6 mg/kg to facilitate endotracheal intubation. Heart rate, mean arterial pressure, and BIS were recorded before and 2 minutes after injection of propofol. Within 24 hours after the operation, the injection site was checked for pain, edema, and allergic reaction by the assessor.

### 2.3. Effect of application of menthol

Sixty patients were included in the second part of this study. As in the first part of the study, the patients were randomly divided into 2 groups in the second part of the study: a menthol group and a solvent group. No premedication was given. After standard monitoring (pulse oximetry, electrocardiogram, and noninvasive arterial pressure) and BIS monitoring had been started, the IV catheter was inserted into the dorsal vein of the left hand in all of the patients without topical anesthesia. Acetated Ringer solution was infused at 200 mL/h. In the menthol group, 1 mL of 30% L-menthol (Sigma and Aldrich Japan Co., Tokyo, Japan) dissolved in 70% ethanol (Nacalai Tesque Inc., Kyoto, Japan) was rubbed on a 5 cm × 15 cm skin area of the dorsum of the left forearm approximately 5 cm from the insertion site of the IV catheter. Thirty percent of menthol was used because it has been suggested that 30% of menthol can induce a cool sensation without skin irritation and redness.^[13]^ In the solvent group, 1 mL of 70% ethanol was rubbed on the skin in the same way as was done in the menthol group. Fifteen minutes after these interventions, 0.5 mg/kg of propofol was injected into the vein through the IV catheter over a period of 5 seconds in both groups. No other analgesics or sedatives were administered before propofol injection.

During propofol injection, the same outcomes were evaluated in the same manner as described for the first part of this study. Briefly, the outcomes evaluated were the incidence of propofol injection-induced pain and the 4-point NRS for propofol injection-induced pain as described above. Assessments were performed by 1 assessor (NW), who had remained blinded to treatment allocation. Patients whose BIS decreased below 80 before pain evaluation were excluded. After evaluation of the NRS scores, induction of anesthesia was continued as described above. Heart rate, mean arterial pressure, and BIS were recorded before and 2 minutes after the injection of propofol. Within 24 hours after the operation, the injection site and the menthol or solvent application site were checked for pain, edema, and allergic reaction by the assessor.

### 2.4. Study outcomes

The primary outcome measure was incidence of propofol injection-induced pain. The secondary outcome measures included severity of propofol injection-induced pain. Exploratory outcomes included blood pressure, heart rate, and adverse effects on the skin.

### 2.5. Statistical analysis

Data for patient characteristics are presented as medians and interquartile ranges or means and standard deviations. The sample size for chi-square test was estimated by using G*Power 3.1. A total sample size of 43 patients was calculated to achieve an effect size of 0.5, with an alpha error of 0.05, and a power of 0.9. To ensure significant power, we decided to include 60 patients in each part of study. Data for patient characteristics were analyzed by Student *t* test. The chi-square test was used for analysis of the incidence of propofol injection-induced pain. The Mann–Whitney *U* test was used for analysis of the NRS scores during IV propofol injection. These analyses were performed using GraphPad Prism version 6.0 (GraphPad Software, San Diego, CA). A *P*-value < .05 was considered as statistically significant.

## 3. Results

### 3.1. Effect of cooling of the skin

There were no significant differences in the data for patient characteristics between the groups (Table 1). There was no patient who felt pain during pretreatment with the cooling gel pad. In the non-cooling group, 1 patient’s BIS decreased below 80 before questioning and the patient’s data were excluded from analysis. The NRS scores during IV injection of propofol in the cooling group and non-cooling group are shown in Table 2. The severity of propofol injection-induced pain, as assessed by the 4-point NRS, was significantly lower in the cooling group (median [IQR]: 0 [0–0.75]) compared to the non-cooling group (1 [0–1], *P* = .027). The incidence of pain in the cooling group (27%) was significantly lower than that in the non-cooling group (52%, *P* = .049). During the first 24 hours after the operation, there was no complication such as pain, edema, or allergic reaction at the injection site. Two minutes after administration of propofol, heart rates were 79 ± 12 beats per minute (bpm) in the cooling group and 77 ± 14 bpm in the non-cooling group, mean blood pressures were 95 ± 14 mm Hg in the cooling group and 100 ± 14 mm Hg in the non-cooling group, and BIS values were 93 ± 4 in the cooling group and 95 ± 4 in the non-cooling group. There were no significant differences in these parameters between the 2 groups.

**Table 1:** Characteristics of patients in the cooling group and non-cooling group.

	Cooling group (n = 30)	Non-cooling group (n = 30)	*P*-value
Age (years)	56 [40–66]	61 [47–67]	.099
Height (cm)	164 (7)	162 (8)	.258
Weight (kg)	62 (10)	59 (10)	.186
Gender (male/female)	17/13	16/14	.795

Values are shown as medians [interquartile ranges], means (standard deviations) or number of patients.

**Table 2:** Numbers of patients with pain during intravenous injection of propofol in the cooling group and non-cooling group.

	Cooling group (n = 30)	Non-cooling group (n = 29)	*P*-value
No pain (0)	22 (73.3)	14 (48.2)	.027
Mild pain (1)	6 (20)	8 (27.5)	
Moderate pain (2)	2 (6.7)	3 (10.3)	
Severe pain (3)	0 (0)	4 (13.8)	
Incidence of pain	8 (26.7)	15 (51.7)	.049

The values in parentheses in the left column represent pain on a 4-point numerical rating scale. Data are shown as number of patients (percentage).

### 3.2. Effect of application of menthol

There were no significant differences in the data for patient characteristics between the groups (Table 3). There was no patient who felt pain during pretreatment with menthol or the solvent. The NRS scores during IV injection of propofol in the menthol group and solvent group are shown in Table 4. The severity of propofol injection-induced pain, as assessed by the 4-point NRS, was significantly lower in the menthol group (0 [0–0]) compared to the solvent group (0 [0–1], *P* = .021). The incidence of pain in the menthol group (13%) was significantly lower than that in the solvent group (40%, *P* = .020). During the first 24 hours after the operation, there was no complication such as pain, edema, or allergic reaction at the injection site or the menthol or solvent application site. Two minutes after administration of propofol, heart rates were 76 ± 12 bpm in the menthol group and 72 ± 10 bpm in the solvent group, mean blood pressures were 96 ± 15 mm Hg in the menthol group and 94 ± 14 mm Hg in the solvent group, and BIS values were 96 ± 2 in the menthol group and 96 ± 2 in the solvent group. There were no significant differences in these parameters between the 2 groups.

**Table 3:** Characteristics of patients in the menthol group and solvent group.

	Menthol group (n = 30)	Solvent group (n = 30)	*P*-value
Age (years)	67 [56–75]	59 [52–70]	.441
Height (cm)	159 (10)	158 (9)	.946
Weight (kg)	59 (12)	58 (12)	.763
Gender (male/female)	17/13	16/14	.795

Values are shown as medians [interquartile ranges], means (standard deviations) or number of patients.

**Table 4:** Numbers of patients with pain during intravenous injection of propofol in the menthol group and solvent group.

	Menthol group (n = 30)	Solvent group (n = 30)	*P*-value
No pain (0)	26 (86.7)	18 (60)	.021
Mild pain (1)	2 (6.7)	6 (20)	
Moderate pain (2)	2 (6.7)	3 (10)	
Severe pain (3)	0 (0)	3 (10)	
Incidence of pain	4 (13.3)	12 (40)	.020

The values in parentheses in the left column represent pain on a 4-point numerical rating scale. Data are shown as number of patients (percentage).

## 4. Discussion

To the best of our knowledge, this is the first study showing the effects of menthol, a selective TRPM8 agonist, on propofol injection-induced pain. The major findings of this study were as follows: (1) skin cooling using a gel pad stored at 7°C lowered skin temperature below 25°C and reduced both the incidence and severity of propofol injection-induced pain; (2) menthol application also reduced both the incidence and severity of propofol injection-induced pain. Although it is difficult to clarify the details of the mechanism in the clinical study, it is significant that our study, in which the effects of cooling stimuli and menthol were examined, showed that TRPM8 channels may be involved in propofol infusion pain.

The following mechanisms can be inferred. First, TRPM8 activation exerts an analgesic effect directly at its channel level and in primary nociceptive afferent neurons. Systemic and topical application of TRPM8 agonists is known to inhibit complete Freund adjuvant-induced inflammatory pain, pain induced by the transient receptor potential ankyrin 1 (TRPA1) agonist acrolein and pain induced by the transient receptor potential vanilloid 1 (TRPV1) agonist capsaicin.^[14–16]^ Second, TRPM8 activation in the periphery leads to the activation of inhibitory neurons in the spinal cord, resulting in analgesia. A previous study suggested that activation of TRPM8-positive afferents leads to central synaptic release of glutamate and inhibits nociceptive afferents or spinal dorsal horn neurons.^[9]^ Additionally, cool stimuli are known to activate C-tactile afferents that can inhibit the activity of spinal dorsal horn neurons involved in nociception.^[17]^ The third possibility is that the non-nociceptive input from the convergent skin area modulates the nociceptive input from deep tissues. Previous studies have shown neural convergence of deep tissues and skin onto common neurons in the spinal dorsal horn.^[18–20]^ The existence of neural convergence suggests that non-nociceptive input from the convergent skin area could modulate the nociceptive input from deep tissues.^[9,21,22]^ Regarding venous nociception, a previous study showed that rubbing the injection site could reduce the intensity of propofol injection-induced pain.^[23]^ Further work will be necessary to clarify the analgesic mechanism of skin cooling and menthol application of propofol injection-induced pain.

Two quantitative systematic reviews evaluated various interventions to prevent propofol injection-induced pain and have recommended injection in the antecubital vein and pretreatment with lidocaine in conjunction with venous occlusion.^[24–26]^ Among the interventions, pretreatment using lidocaine with venous occlusion was the most effective method to prevent propofol injection-induced pain (relative risk [RR] 0.29, 95% confidence interval [95% CI] 0.22–0.38).^[24]^ Other effective interventions included opioids (RR 0.49, 95% CI 0.41–0.59), ketamine (RR 0.52, 95% CI 0.46–0.57), and non-steroidal anti-inflammatory drugs (RR 0.67, 95% CI 0.49–0.91).^[24]^ However, the incidence of propofol injection-induced pain in patients who received lidocaine pretreatment, was 13% to 32%,^[25,26]^ and IV injection of lidocaine itself causes pain.^[13]^ In the present study, the incidences of propofol injection-induced pain in the cooling group and menthol group were 27% and 13%, respectively. These results suggest that topical application of menthol may be more effective than skin cooling for reducing propofol injection-induced pain. The incidence of propofol injection-induced pain in the menthol group was comparable to that in patients treated with lidocaine in previous studies.^[13,24]^ Both interventions evaluated in this study are simple, safe, and effective methods for reducing the incidence and severity of propofol injection-induced pain. Notably, no patient experienced severe pain in either the skin cooling or menthol groups. Additionally, these topical methods can be combined safely with other interventions, such as IV administration of lidocaine.

This study has several limitations. Firstly, this study was conducted using a single-blind design in which the pain assessors were blind to group allocation, but the anesthesiologists and participants were not. Since the study protocol involved placing a cooling gel pad on the skin of conscious participants for 15 minutes or administering menthol dissolved in a solvent onto their skin, it was difficult to adopt a double-blind study design. To minimize the effects of the bias, a room temperature gel pad and solvent were used for the control group. These approaches reflect efforts to control for confounding factors other than temperature and TRPM8 agonists as much as possible. However, mechanical stimulation from the gel pad was present in the cooling group, and solvent-related effects were present in the menthol group. Therefore, a direct comparison between the cooling and menthol groups is not feasible. Secondly, since the concentration of menthol and the temperature in deep tissues are uncertain, we could not conclude whether the analgesic effects of menthol and skin cooling are associated with activation of inhibitory neurons via activating TRPM8 on the skin or direct activation of TRPM8 located in the vein. Since the basement membrane acts as a diffusion barrier that prevents topically applied drugs from reaching subcutaneous receptors,^[27]^ topical application of menthol would mainly activate TRPM8 on the skin and induce an analgesic effect on propofol injection-induced pain.

## 5. Conclusion

The results of this study showed that cooling and application of menthol are effective for reducing pain experienced during IV injection of propofol.

## Author contributions

**Conceptualization:** Takashi Ishida.

**Data curation:** Natsuko Watanabe.

**Investigation:** Takashi Ishida, Natsuko Watanabe.

**Software:** Takashi Ishida.

**Writing – original draft:** Takashi Ishida.

**Writing – review & editing:** Satoshi Tanaka, Mikito Kawamata.
